# Accidental displacement and migration of endosseous implants into adjacent craniofacial structures: A review and update

**DOI:** 10.4317/medoral.18032

**Published:** 2012-05-01

**Authors:** Alberto González-García, Jaime González-García, Marcio Diniz-Freitas, Abel García-García, Pedro Bullón

**Affiliations:** 1 DDS, PhD. Assistant Professor, Department of Stomatology, School of Dentistry, University of Seville, Spain; 2 MD, PhD. Physician Specialist. Otolaryngology Departament, University Hospital “Virgen Macarena”, Seville, Spain; 3 DDS, PhD. Assistant Professor, Department of Stomatology, School of Dentistry, University of Santiago de Compostela, Spain; 4 MD, PhD. Head of Section. Department of Maxillofacial Surgery, Clinical University Hospital, University of Santiago de Compostela, Spain; 5 MD, PhD. Professor of Periodontics, School of Dentistry, University of Seville, Spain

## Abstract

Objectives: Accidental displacement of endosseous implants into the maxillary sinus is an unusual but potential complication in implantology procedures due to the special features of the posterior aspect of the maxillary bone; there is also a possibility of migration throughout the upper paranasal sinuses and adjacent structures. The aim of this paper is to review the published literature about accidental displacement and migration of dental implants into the maxillary sinus and other adjacent structures. Study Design: A review has been done based on a search in the main on-line medical databases looking for papers about migration of dental implants published in major oral surgery, periodontal, dental implant and ear-nose-throat journals, using the keywords “implant,” “migration,” “complication,” “foreign body” and “sinus.” Results: 24 articles showing displacement or migration to maxillary, ethmoid and sphenoid sinuses, orbit and cranial fossae, with different degrees of associated symptoms, were identified. Techniques found to solve these clinical issues include Cadwell-Luc approach, transoral endoscopy approach via canine fossae and transnasal functional endoscopy surgery. Conclusion: Before removing the foreign body, a correct diagnosis should be done in order to evaluate the functional status of the ostiomeatal complex and the degree of affectation of paranasal sinuses and other involved structures, determining the size and the exact location of the foreign body. After a complete diagnosis, an indicated procedure for every case would be decided.

** Key words:**Implant, oral surgery, foreign body, paranasal sinuses, displacement, migration.

## Introduction

In the last two decades, the osseointegrated rehabilitation for partial or complete edentulous patients has become a routine practice in the medical society, having predictable results on the long term (1), and these techniques have become increasingly widespread. Sometimes the particular conditions of the alveolar ridges in which these endosseous implants are going to be placed may be unfavorable. The main adverse condition is bone atrophy following natural teeth loss; moreover, the atrophy of the residual alveolar bone is chronic, progressive, cumulative and irreversible (2).

New surgical techniques to reconstruct the alveolar ridges have arisen in the last decades because of the increasing demand and the unfavorable specific conditions of the alveolar bone. This is the case of different types of bone grafts at the maxillary and mandibular level, alveolar osteogenesis distraction and maxillary sinus floor elevation (3). The aim of all these techniques is focused at achieving a better volume of available bone in order to insert long-length implants with a more favorable crown-to-implant ratio (4).

Rehabilitating the posterior maxillary area often becomes a challenge for the oral surgeon for three reasons: reabsorption of the edentulous alveolar ridge; progressive pneumatization of the maxillary sinuses; and low density of the mandibular bone at that level, which normally corresponds to a type IV bone, according to Cawood and Howell’s classification (5). Although the elevation of the maxillary sinuses with bone grafts is a high predictability technique in terms of successful osseointegrated rehabilitation (6), sometimes it is not used for different reasons, conditioning the insertion of short-length implants or with poor stability at this level.

The insertion of implants without an adequate primary stability or with a lack of osseointegration at this level, can frequently lead to accidental displacement into the maxillary sinus. This complication requires a correct management and, if this surgical complication is not treated adequately, the implant can migrate to upper craniofacial structures causing surgical complications due to foreign-body reactions, infection and tissue necrosis and the collapse of the sinus clearance.

The aim of this paper is to review the published literature on accidental displacement and migration of dental implants into the maxillary sinus and other adjacent structures.

## Material and Methods

This review is based on a search into the main on-line medical databases looking for papers about migration of dental implants published in major oral surgery, periodontal, dental implant and ear-nose-throat (ENT) journals between January 1970 and January 2011, using the keywords “implant,” “migration,” “complication,” “foreign body” and “sinus.” Other relevant papers were identified in the references sections of papers retrieved by the primary search.

## Results

24 articles were identified. The majority of them are related to accidental displacement and migration of dental implants to maxillary sinus (7-26). The search showed that migration has also been described in other craniofacial structures such as the ethmoid sinuses (27), sphenoid sinuses (28), orbit (24,29) and cranial fossae (30) ( Table 1Surgical techniques to rescue these implants differ depending on the criteria of the authors, related associated symptoms and location. The found techniques to remove implants displaced to maxillary sinus includes the conservative management with periodical revisions, the Cadwell-Luc approach breaking the sinus membrane, the transoral endoscopy approach via canine fossae and transnasal functional endoscopy sinus surgery (FESS). This last technique is used to remove implants displaced to maxillary sinus as well as to remove implants migrated up to upper structures.

**Table 1:**
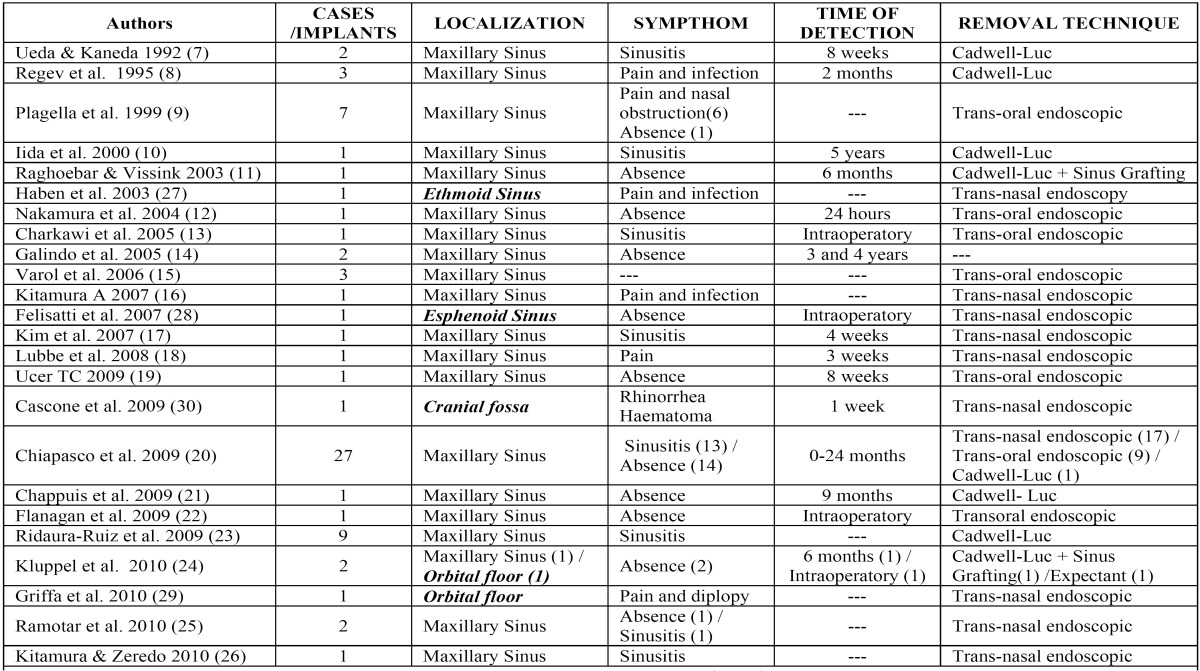
Reported cases of displaced and migrated endosseous implants in the literature.

## Discussion

Among all maxillary sinusitis surgically treated, around 5-15 % are caused by foreign body of dental origin (31,32). The typical bodies described are: dental roots, impression materials, endodontic material and amalgam. However, dental implants have become a new common foreign body in the last years since implantology surgery has become a routine surgical procedure in dental clinics due to the increasing demand. This technique has led to surgical complications that dentists, oral surgeons and ENT surgeons should be aware of for their correct diagnosis and management.

As the displacement of foreign bodies of dental origin into paranasal sinuses can be followed by complications, including sinusitis or aspergillosis (33-35); early removal of the implants displaced into the sinuses is advisable as well as it occurs with other metallic foreign bodies, in order to prevent the development of both physical and chemical chronic irritation that can lead to neoplastic conditions (36).

First of all, there are accidental intraoperatories displacements towards the maxillary sinuses associated with overtreatment of the implant preparation, poor primary stability or worse planning. In these cases, the displacement occurs as a surgical complication, disappearing across the maxillary during the implant preparation.

On the other hand there are also migration of implants into the maxillary sinuses that can occur years after the placement. In these cases, the phenomenon that leads to the migration is unknown. The possible mechanisms that could explain migration of an implant into the maxillary sinus were inflammatory reaction causing periimplantitis or bone reabsorption caused by an incorrect distribution of occlusal forces (14).

Finally, accidentally displaced implants can also migrate from the maxillary sinus reaching upper structures by physiological sinus clearance against gravitational force in most cases and, in other cases by means of intra-nasal changes of pressure and due to foreign body reaction and local tissue necrosis. The involved structures by migrations can be other paranasal sinuses, orbital floor or cranial fossa.

The maxillary sinus is the most common location described in the literature and the removal of implants can be performed using a direct approach through the oral mucosa (10,12); transorally by endoscopy (9,15) or transnasally by sinus endoscopy approach (16,17). In other locations described such as ethmoid and sphenoid sinuses, orbit and cranial fossae, the migrated implant has been removed by transnasal functional endoscopic approach in all cases (27-30). (Table 1)

The majority of authors describe different degrees of pathological changes in the sinuses where the implants are located, usually due to deficiencies in the sinus clearance (12-16,27,28).

**Figure 1:**
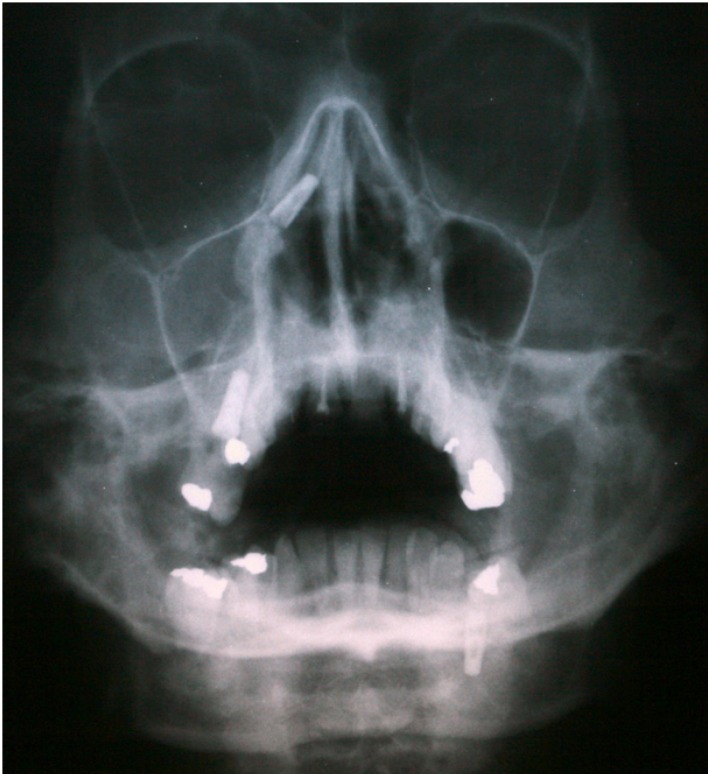
Oblique frontal cephalogram showing upper metallic foreign body and right maxillary sinus total occupation.

**Figure 2:**
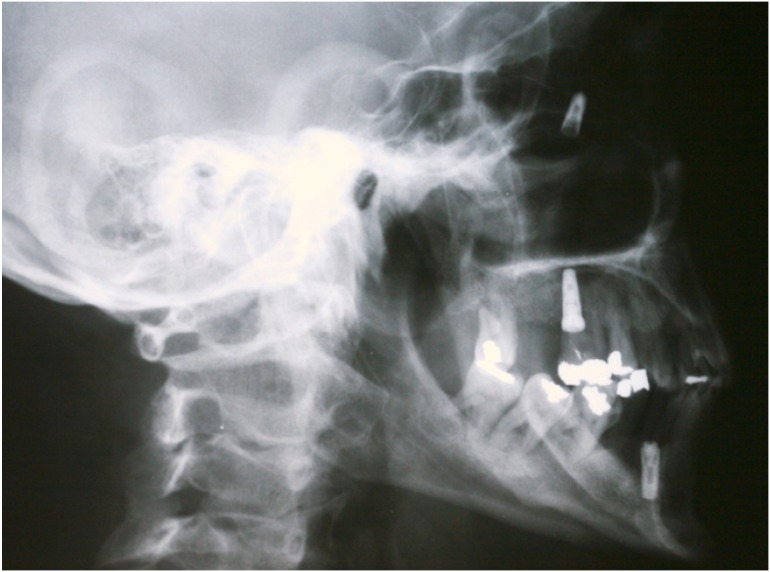
Lateral cephalogram showing an endosseous implant.

**Figure 3:**
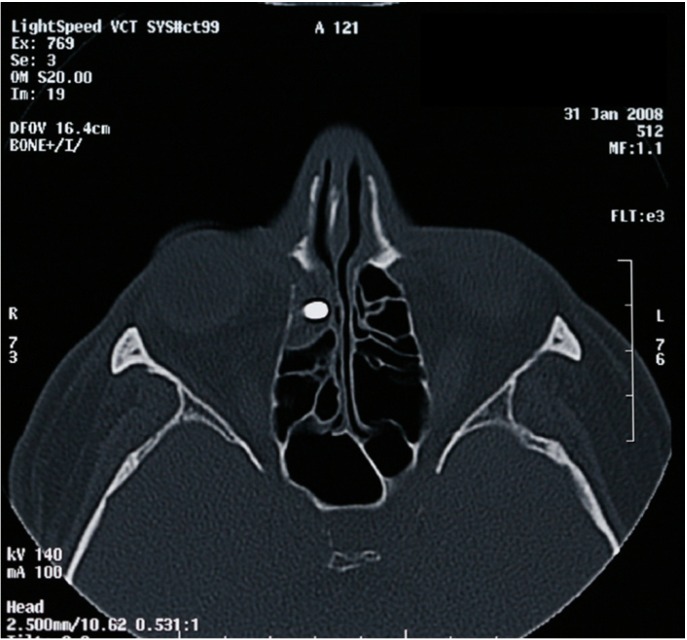
Coronal computerized tomography showing exophthalmia and partial occupation of the ethmoid sinuses by a metallic foreign body (an endosseous implant).

The correct diagnosis is based on: history of previous implantology surgery, clinical exploration and radiological findings. Radiological techniques show location and size of the metallic foreign body, and so panoramic radiography is commonly used at dental clinics; however, with lateral and oblique frontal cephalograms (Fig. 1,2) and computerized tomography (Fig. 3) the exact location of the foreign body is better demonstrated, as well as the degree of structural damage and sinus occupation, which are necessary for the correct treatment.

Endoscopy removal of implants brought some interest a few years ago, due to its low morbidity, rapid recovery period and possibility of treating the affected paranasal sinuses. As compared with other approaches, transnasal endoscopy removal of foreign bodies migrated into paranasal sinuses allows not only the removal of the implant, but also a simultaneous treatment of hyperplasic, hypertrophic or infected mucosa. Such treatment has proven to be less aggressive and, moreover, it preserves the mucociliary integrity and function reducing the overall treatment and recovery time. Besides, it allows the treatment of natural maxillary ostium when needed (25,26). If the problem extends to more than one paranasal sinus, the endoscopy approach allows the simultaneous treatment of the other affected sinuses. Another advantage of the endoscopy approach is that it could be performed under local anesthesia and sedation, contributing to a lower morbidity rate and a shorter recovery period (37).

Meanwhile, the Caldwell-Luc approach it would only be indicated when neither the osteomeatal complex nor other paranasal sinuses are affected, and it would be reserved as a first election technique when the foreign body has a considerable size ( Table 2) and it is not available by means of endoscopy (38). Nevertheless, in some cases the Caldwell-Luc approach seems to be highly effective in the treatment of refractory chronic sinusitis after a failed endoscopy approach and it should be considered as a viable technique (39). For the same Caldwell-Luc approach, Raghoebar & Vissink (11) and Kluppel et al. (24) report that, in case there is no pathology associated with the migration of the implant into the maxillary sinus, the approach should be direct removing the implant in combination with a bone graft, in order to increase the volume of the alveolar ridge, thus saving the patient from surgery and reducing the total recovery time. In the same way, Ucer described a case using a transnantral endoscopy approach combined with simultaneous sinus grafting (19).

**Table 2:**
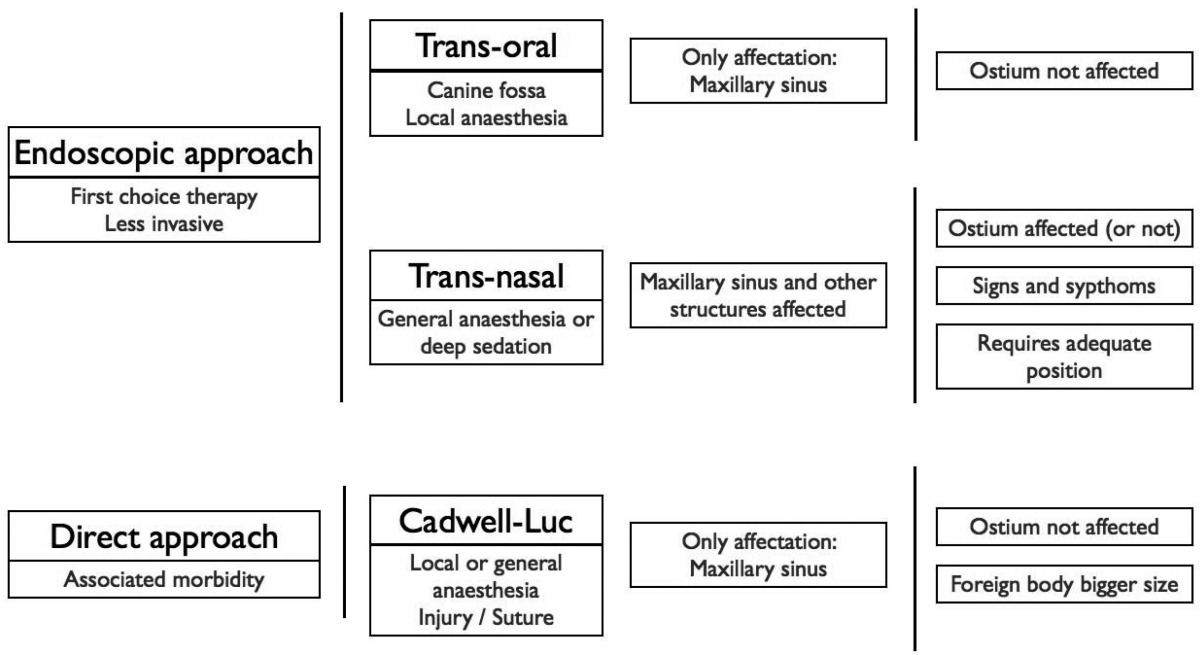
Therapeutic options for approach and removal of endosseous implants as foreign bodies from paranasal sinuses and adjacent structures.

First of all and in order to avoid future complications, when the bone volume is inadequate to receive an implant with a proper length and stability, the possibility of a maxillary sinus floor augmentation using a bone graft can be useful to improve bone quality and bone volume. Despite placement of endosseous implants - with or without previous bone grafts - has a reasonably good prognosis, (6) a minimum length and width should be considered (8) as well as the alternative of angulated implants, when possible. It is appropriate to remark that an adequate morphological implant design might prevent this sort of complications and a blind attempt to capture and extract the displaced implant may cause an unnecessary widening of an alveolar preparation and could damage the lining of the maxillary sinus (15).

Secondly, following the same prevention line, if the implant does not provide enough primary stability at this level and its mobilization is possible, it should be removed in order to avoid immediate displacements or delayed migrations.

When an implant is inserted accidentally into the maxillary sinus it should be removed as soon as possible to avoid further complications such as facial pain, airway obstruction, nasal discharge and infection triggered by the possible migration into other upper structures as it has been shown in this paper. In the view of this, conservative management is not recommended.

On the other hand and in opposition to what it could be thought, the Caldwell-Luc approach is not the first choice procedure to solve this surgical accident, despite being the best known technique by oral surgeons ( Table 2). Foremost, before removing the foreign body, a diagnostic endoscopy should be performed to evaluate the functional status of the ostiomeatal complex, and an interconsult to an ENT Unit should be appropiate. After diagnosing the degree of affectation of the paranasal sinuses, determining the size and the exact location of the foreign body, the indicated procedure for every case would be decided. If the structure of the ostiomeatal complex appears to be normal, it is possible to limit the procedure to transoral endoscopy sinus surgery through the canine fossa. A minimum of local anesthetic is needed and it does not require suturing, in contrast to the classical Caldwell-Luc approach. If there is any disorder of the ostiomeatal complex or there is affection in other paranasal sinus, the case must be treated endoscopically with a transnasal approach, by means of transnasal meatostomy and the removal of the foreign body. In these cases, as well as when migration to upper structures occurs, the transnasal functional endoscopy surgery is a minimally invasive technique, with low morbidity levels that allows access and cleaning of the affected sinuses.
